# Ulcération cutanée: penser aux métastases

**DOI:** 10.11604/pamj.2017.28.304.13326

**Published:** 2017-12-11

**Authors:** Youssef Zemmez, Badreddine Hassam

**Affiliations:** 1 Service de Dermatologie, CHU Ibn Sina Rabat, Maroc

**Keywords:** Ulcérations cutanées, métastases cutanées, cancer pulmonaire, Cuteneous ulcers, cutaneous metastases, lung cancer

## Abstract

We report the case of a 68-year old patient, with a history of chronic smoking, presenting with cutaneous swelling at the level of the anterior chest wall evolving over a period of 5 months and gradually increasing in size. It was associated with gradually worsening exertional dyspnoea evolving in a context of an alteration of general state and 15kg weight loss. Mucocutaneous examination objectified rounded, well-defined cutaneous swelling measuring 5cm x 5cm, with erythematous border and with ulcerated surface (A). Lymph nodes examination showed two painless, mobile, bilateral axillary adenopathies with a firm consistency, each measuring 2cm. Histological and immuno-histochemical examination of skin biopsy specimen showed adenocarcinoma compatible with a lung origin (B and C). Chest CT scan confirmed the presence of left lingular pulmonary process (D). The therapeutic approach was based on pemetrexed + platinum salt chemotherapy. The patient died 4 months after the diagnosis of the disease.

## Image en médecine

Patient de 68 ans, tabagique chronique, consultait pour une tuméfaction cutanée au niveau de la paroi thoracique antérieure évoluant depuis 05 mois, augmentant progressivement de taille, associée à une dyspnée d'effort d'aggravation progressive, évoluant dans un contexte d'altération de l'état général et d'amaigrissement chiffré à 15kg. L'examen cutanéo-muqueux objectivait Une tuméfaction cutanée mesurant 5cm sur 5cm, arrondie, bien limitée, à bordure érythémateuse et à surface ulcérée (A). L'examen des aires ganglionnaires montrait deux adénopathies axillaires bilatérales, mesurant 2cm chacune, de consistance ferme, indolores, mobiles. La biopsie cutanée montrait un profil histologique et immuno-histochimique d'un adénocarcinome compatible avec une origine pulmonaire (B et C). La TDM thoracique avait confirmé la présence d'un processus pulmonaire lingulaire gauche (D). La décision thérapeutique était de démarrer une poly-chimiothérapie à base de sel de platine et pemetrexed. L'évolution était marquée par le décès du malade après 4 mois du diagnostic de la maladie.

**Figure 1:**
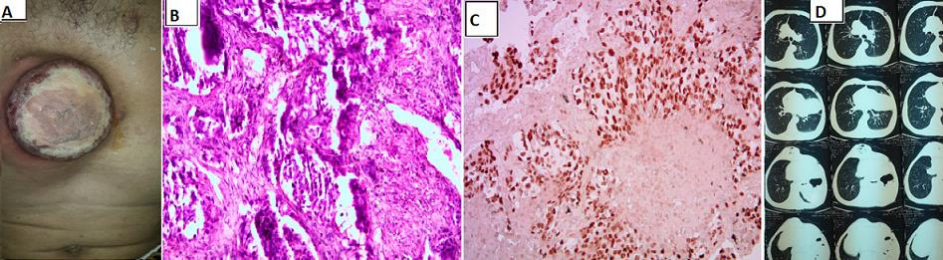
(A) ulcération cutanée de la paroi thoracique antérieure; (B) histologie en faveur d’une métastase; (C) immunohistochimie: TTF 1+; (D) TDM thoracique: processus pulmonaire lingulaire gauche

